# Long-term results after mitral valve surgery using minimally invasive versus sternotomy approach: a propensity matched comparison of a large single-center series

**DOI:** 10.1186/s12872-021-02121-3

**Published:** 2021-06-26

**Authors:** Ayse Cetinkaya, Anna Geier, Karin Bramlage, Stefan Hein, Peter Bramlage, Markus Schönburg, Yeong-Hoon Choi, Manfred Richter

**Affiliations:** 1 Department of Cardiac Surgery, Kerckhoff-Heart Center Bad Nauheim, Justus-Liebig University Giessen, Benekestraße 2-8, 61231 Bad Nauheim, Germany; 2 Institute for Pharmacology and Preventive Medicine, Cloppenburg, Germany

**Keywords:** Mitral valve, Minimally invasive mitral valve surgery, Mitral valve repair, Sternotomy, Outcomes

## Abstract

**Background:**

Mitral valve (MV) surgery has traditionally been performed by conventional sternotomy (CS), but more recently minimally invasive surgery (MIS) has become another treatment option. The aim of this study is to compare short- and long-term results of MV surgery after CS and MIS.

**Methods:**

This study was a retrospective propensity-matched analysis of MV operations between January 2005 and December 2015.

**Results:**

Among 1357 patients, 496 underwent CS and 861 MIS. Matching resulted in 422 patients per group. The procedure time was longer with MIS than CS (192 vs. 185 min; *p* = 0.002) as was cardiopulmonary bypass time (133 vs. 101 min; *p* < 0.001) and X-clamp time (80 vs. 71 min; *p* < 0.001). ‘Short-term’ successful valve repair was higher with MIS (96.0% vs. 76.0%, *p* < 0.001). Length of hospital stay was shorter in MIS than CS patients (10 vs. 11 days; *p* = 0.001). There was no difference in the overall 30-day mortality rate. Cardiovascular death was lower after MIS (1.2%) compared with CS (3.8%; OR 0.30; 95%CI 0.11–0.84). The difference did not remain significant after adjustment for procedural differences (aOR 0.40; 95%CI 0.13–1.25). Pacemaker was required less often after MIS (3.3%) than CS (11.2%; aOR 0.31; 95%CI 0.16–0.61), and acute renal failure was less common (2.1% vs. 11.9%; aOR 0.22; 95%CI 0.10–0.48). There were no significant differences with respect to rates of stroke, myocardial infarction or repeat MV surgery. The 7-year survival rate was significantly better after MIS (88.5%) than CS (74.8%; aHR 0.44, 95%CI 0.31–0.64).

**Conclusion:**

This study demonstrates that good results for MV surgery can be obtained with MIS, achieving a high MV repair rate, low peri-procedural morbidity and mortality, and improved long-term survival.

## Background

Minimally invasive surgery (MIS) has evolved as an alternative to conventional sternotomy (CS) when performing mitral valve (MV) surgery. In Germany, slightly more than half (55%) of patients undergoing MV surgery are treated using an MIS approach [1]. MIS techniques were introduced to reduce surgical trauma and postoperative recovery time [2]. Potential benefits of MIS techniques include less postoperative bleeding, fewer wound infections, faster postoperative recovery, shorter intensive care unit (ICU) and/or hospital stay, and the cosmetic benefits of a smaller scar [3–9]. Meta-analyses of the available evidence also suggest that perioperative mortality, repair and reoperation rates after MIS are at least similar to those seen after CS or better [3–9]. Few randomised controlled trials have been performed and meta-analysed [3] and most of the evidence comes from retrospective observational studies [6]. In addition, most of these studies evaluated only short- to mid-term outcomes [3–9].

The risk profiles of patients selected for MIS often differ from those treated via CS, with the latter approach favoured for higher risk patients, making it difficult to compare outcomes for the two approaches. Propensity score matching is usually performed to control for such differences. Analyses comparing MIS and CS that use this method generally support the findings of other studies and meta-analyses [10–20]. Only a few propensity-matched studies have evaluated long-term outcomes, with most finding similar long-term survival after MIS and CS [12, 18, 20], although one reported better 5- and 10-year survival rates with MIS among patients with degenerative MV regurgitation [13].

The purpose of our investigation, therefore, was to compare and corroborate the short- and long-term results of MV interventions after CS and MIS at our center.

## Methods

This study, performed between January 2005 and December 2015, was a single-center, retrospective, propensity score matched analysis of MV surgeries. Approval for the study was obtained from the site’s ethical committee and it complied with the Declaration of Helsinki and its amendments. Written informed consent from patients was not required because the study used anonymised data that had already been collected as part of routine diagnosis and treatment.

### Patient population

Patients undergoing isolated MV surgery, or MV surgery combined with tricuspid valve repair and ablation therapy, patent foramen ovale (PFO) or atrial septal defect (ASD) closure, within the period January 2005–December 2015 were included in the study. Patients were excluded from the study if they underwent a concomitant coronary artery bypass graft (CABG), an aortic valve procedure, an intervention of the ascending aorta or if they had severe pulmonary adhesion or severe calcification of the mitral annulus.

### Group assignment

The procedures were performed by a total of 5 surgeons over this time period with no particular preference for either intervention. All surgeons were qualified to perform both procedures and only some patient selection criteria as outlined above and the time of intervention (more CS in the first few years of the study starting 2005 and more MIS later on in the study) resulted in group assignment. MIS patients requiring conversion to open surgery remained assigned to the MIS group.

We propensity matched the available patients resulting in 422 matched patients per group. The propensity score for each patient was calculated by logistic regression and was adjusted for 15 key baseline variables. These comprised age, gender, hypertension, dyslipidemia, New York Heart Association (NYHA) score ≥ III, Canadian Cardiac Society (CCS) score ≥ III, log EuroScore I, pulmonary hypertension, prior aortic valve replacement, prior CABG, emergency indication, MV pathology, prior pacemaker implantation, renal insufficiency and left ventricular ejection fraction [LVEF]. When matching patients between the two groups, a difference in propensity score of 5% (0.05) was tolerated.

### Surgical procedure

The CS approach was performed using full median sternotomy, cannulation of the ascending aorta and both vena cavae, and antegrade crystalloid (Bretschneiders) cardioplegia. MV exposure was obtained through the interatrial (Waterston) groove. MIS was performed with regular endotracheal intubation. Cardiopulmonary bypass (CPB) was accomplished by femoro-femoral cannulation, under transoesophageal echocardiographic (TEE) monitoring. In cases of femoral artery obstruction, cannulation of the right axillary artery was used. Patients with concomitant tricuspid valve disease and patients above 100 kg bodyweight received a second venous cannula inserted percutaneously into the right jugular vein. Vacuum assisted CPB and moderate hypothermia (34 °C) were used. Surgical access to the MV was obtained using right lateral minithoracotomy in the fourth intercostal space, a soft tissue retractor and eventually a metal spreader. Direct aortic cross-clamping was accomplished with the Chitwood clamp and video assistance was used. Carbon dioxide field flush was applied, antegrade crystalloid (Bretschneiders) cardioplegia was administered, left atriotomy was performed in the Waterston groove, and a specific left atrial retractor was used. In all patients TEE was used for immediate quality control.

### Data, outcomes and definitions

A review of all electronic medical records for patients who had undergone an MV procedure was undertaken. Medical records detailed clinical variables including patient age, sex, comorbid diseases, prior cardiology procedures, echocardiographic procedures and other pertinent medical/surgical history. Mitral insufficiency was graded as grade I (mild regurgitation < 20%), grade II (moderate; 20–40%), grade III (moderate to severe; 40–60%) or grade IV (severe; > 60% regurgitant fraction). Pulmonary hypertension was based on a threshold of > 60 mmHg, as defined in the EuroSCORE I. Patient risk was determined using EuroSCORE, which incorporates a number of variables to define the patient’s risk level. At the patient’s last follow-up hospital visit, data was collected on complications and echocardiography parameters.

### Statistics

Data were analysed using descriptive statistics. Categorical variables are presented as absolute values and frequencies (%), while mean with standard deviation (SD) or median and interquartile range (IQR) were used for continuous variables. T-tests and Mann–Whitney U-tests were used for continuous variables to make comparisons between the MIS and CS groups, while Fisher’s exact or Chi-square tests were used for comparisons of categorical variables. Kaplan–Meier curves were used for survival analyses. Hazard ratios (HR) were calculated using Cox regression. Odds ratios (OR) were calculated by logistic regression and adjusted for procedural differences between the two groups (MV repair, MV replacement, cryoablation, left atrial appendage closure [LAA] and concomitant tricuspid valve repair [TVR]). A two-tailed p-value of < 0.05 was considered to be statistically significant. We used IBM SPSS Statistics software version 24.0 (IBM Corporation, Armonk, NY, USA) for all statistical tests.

## Results

**Fig. 1:**
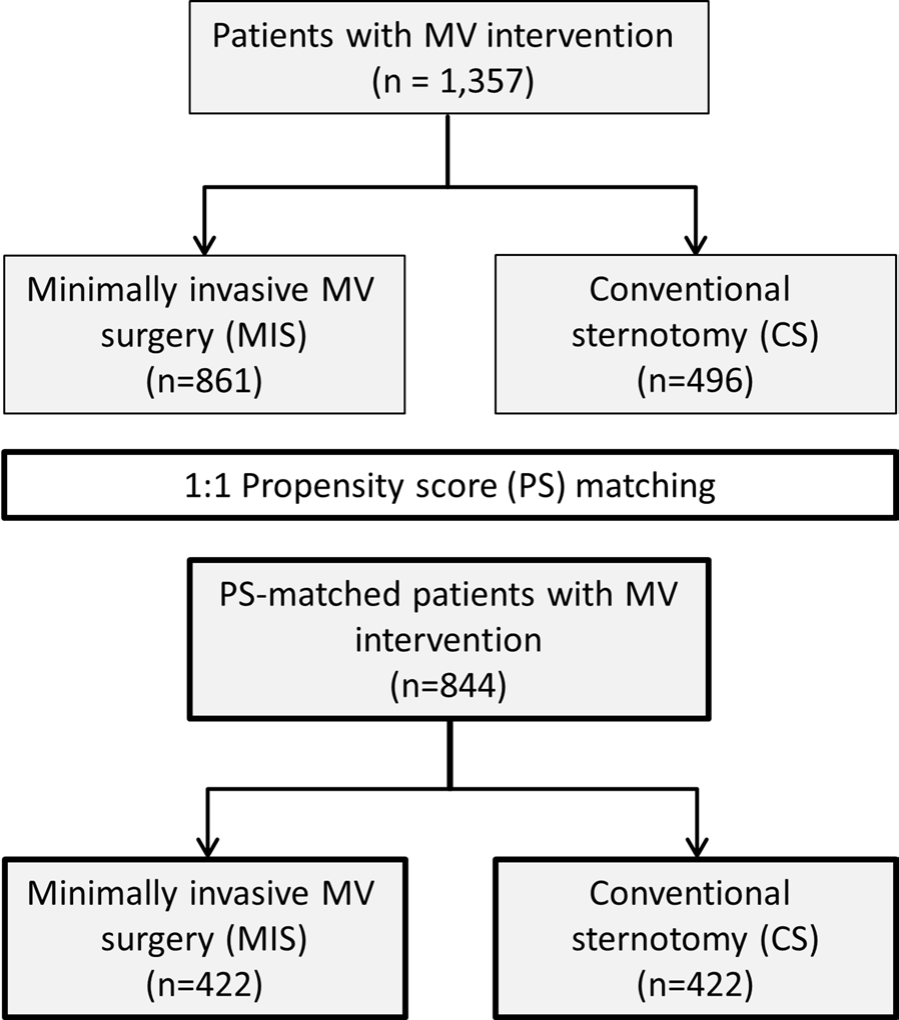
Flow chart. *CS* conventional sternotomy, *MIS* minimally invasive MV surgery, *MV* mitral valve, *PS* propensity score

The disposition of patients is summarised in Fig. 1. Among 1357 patients who received an MV intervention, 861 were treated using MIS and 496 underwent CS. Propensity score matching resulted in 422 MIS patients and 422 CS patients for analysis.

### Patient characteristics

While there were a number of significant imbalances of baseline characteristics for the overall (unmatched) population (Table 1), the only difference between the matched groups was a slightly higher mean body mass index (*p* = 0.026) in the CS group compared with the MIS group.

**Table 1:** Patient characteristics

	Unmatched				Matched		
	Total N = 1357	MIS N = 861	CS N = 496	*p* value	MIS N = 422	CS N = 422	*p* value
	Mean ± SD or %	Mean ± SD or %	Mean ± SD or %		Mean ± SD or %	Mean ± SD or %	
Age in years	63.9 ± 12.3	63.2 ± 12.2	65.2 ± 12.6	**0.003**	63.7 ± 12.6	64.5 ± 12.8	0.383
Female gender	43.3	41.6	46.4	0.086	45.3	45.3	1.000
BMI (kg/m^2^)	26.4 ± 4.7	26.2 ± 4.5	26.5 ± 4.9	0.315	25.8 ± 4.3	26.5 ± 4.8	**0.026**
CV risk factors							
Hypertension	53.7	56.4	49.0	**0.008**	48.8	50.2	0.680
Dyslipidemia	16.8	22.6	6.9	**< 0.001**	8.8	7.6	0.530
Comorbidities general							
Diabetes mellitus	8.7	8.4	9.3	0.566	6.4	9.5	0.098
Kidney failure (Crea. > 2.26 mg/dL)	1.7	0.6	3.6	**< 0.001**	0.9	1.7	0.363
Stroke	5.5	5.5	5.4	0.991	5.9	4.5	0.353
COPD	11.8	12.1	11.3	0.664	12.3	10.4	0.386
PAD	3.0	2.7	3.6	0.321	2.1	2.4	0.817
Comorbidities cardiac							
Atrial fibrillation	32.4	32.5	32.3	0.919	35.3	31.5	0.243
Coronary artery disease	10.2	9.6	11.3	0.334	6.9	8.5	0.366
Prior MI (≤ 90 days)	0.7	0.8	0.4	0.500	1.2	0.5	0.451
Prior Aortic Valve Replacement	2.1	1.5	3.0	0.059	2.1	1.7	0.614
Prior CABG	3.9	3.0	5.4	**0.026**	2.4	3.6	0.310
Prior Pacemaker	2.2	1.6	3.2	0.051	1.9	2.1	0.806
NYHA class III/IV	75.5	69.0	86.9	**< 0.001**	85.1	84.6	0.848
CCS class III/IV	4.1	2.2	7.3	**< 0.001**	4.0	3.8	0.859
Pulmonary hypertension	12.0	14.8	7.3	**< 0.001**	9.0	8.1	0.622
Emergency indication for surgery	3.9	5.0	2.0	**0.006**	2.1	1.9	0.806
Log EuroSCORE I	7.8 ± 10.7	6.7 ± 10.2	9.7 ± 11.5	**< 0.001**	7.3 ± 11.4	7.9 ± 9.1	0.422

*p* values that are statistically significant are highlighted in bold

*BMI* body mass index, *CABG* coronary artery bypass graft, *CCS* Canadian Cardiovascular society, *COPD* chronic obstructive pulmonary disease, *CV* cardiovascular, *MI* myocardial infarction, *NYHA* New York Heart Association, *PAD* peripheral artery disease, *SD* standard deviation

### Mitral valve pathology

**Fig. 2:**
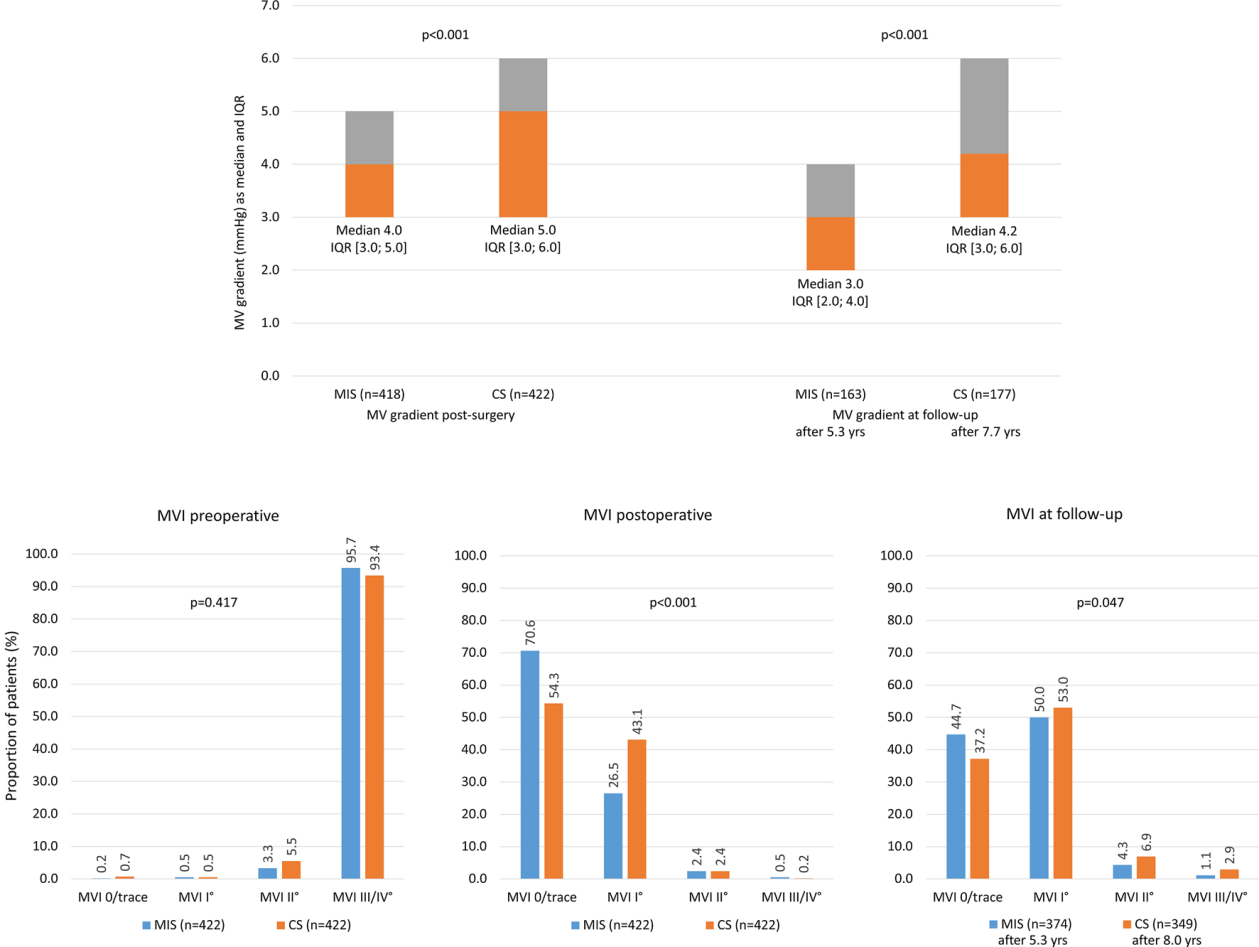
Mitral valve (MV) gradient and competency (mitral valve insufficiency; MVI). *CS* conventional sternotomy, *IQR* interquartile range, *MIS* minimally invasive MV surgery, *MV* mitral valve, *MVI* mitral valve insufficiency

Table 2 summarises MV pathology and echocardiographic findings for the matched MIS and CS cohorts. Mean left and right atrial diameters were greater in the MIS group than in the CS group (both *p* < 0.001). The vena contracta diameter was also greater in the MIS group than the CS group (*p* = 0.002). The only significant differences in terms of MV pathology were higher rates of annulus dilatation (*p* < 0.001) and posterior mitral valve leaflet (PML) prolapse (*p* < 0.001) in the MIS group compared with the CS group. Severe (grade III/IV) mitral regurgitation was present in 95.7% of the MIS group and 93.4% of the CS group (*p* = 0.417; Fig. 2b).

**Table 2:** MV pathology and echocardiographic parameters

	MIS (N = 422)		CS (N = 422)		
	n or n/N	% or Median (IQR)	n or n/N	% or Median (IQR)	*p* value
Echocardiographic parameters					
LVEF (%)	422	60.0 (55.0–63.0)	422	55.0 (53.0–62.0)	0.044
LVEDD (mm)	384	55.0 (50.3–58.0)	308	55.0 (51.0–60.0)	0.518
LVESD (mm)	368	35.0 (31.0–40.0)	303	35.0 (31.0–41.0)	0.363
Left atrial diameter (mm)	385	56.0 (48.0–64.0)	307	49.0 (44.0–58.0)	**< 0.001**
Right atrial diameter (mm)	384	47.0 (40.0–54.0)	306	41.5 (35.0–49.0)	**< 0.001**
Mitral opening (mm)	54	3.6 (2.8–4.6)	175	3.8 (3.1–4.8)	0.242
PISA radius (mm)	85	1.2 (1.0–1.4)	53	1.0 (1.0–1.3)	0.199
Vena contracta (mm)	168	7.0 (5.0–8.0)	114	6.0 (5.0–7.0)	**0.002**
MV pathologies					
Degenerative	399/422	94.5	393/422	93.1	0.390
Functional	23/422	5.5	29/422	6.9	0.390
Acute endocarditis	16/422	3.8	23/422	5.5	0.251
Annulus dilatation	400/422	94.8	366/422	86.7	**< 0.001**
Annulus calcification	29/422	6.9	43/422	10.2	0.085
AML prolapse	87/422	20.6	99/422	23.5	0.319
AML flail	27/422	6.4	29/422	6.9	0.782
PML prolapse	289/422	68.5	224/422	53.1	**< 0.001**
PML flail	182/422	43.1	184/422	43.6	0.890
Chordae elongation	107/422	25.4	90/422	21.3	0.167
Restrictive leaflet	45/422	10.7	56/422	13.3	0.243
MV stenosis	22/422	5.2	31/422	7.3	0.202
MV insuff. ≥ grade II	418/422	99.1	417/422	98.8	1.000

*p* values that are statistically significant are highlighted in bold

*AML* anterior mitral valve leaflet, *LVEDD* left ventricular end-diastolic pressure, *LVEF* left ventricular ejection fraction, *LVESD* left ventricular end-systolic pressure, *MI* myocardial infarction, *MV* mitral valve, *PISA* proximal isovelocity surface area, *PML* posterior mitral valve leaflet, *SD* standard deviation

### Mitral valve surgery

MV repair was performed more often than valve replacement in both the MIS and CS groups. MV repair was performed in 89.6% of patients who underwent MIS compared with 83.9% of those who underwent CS (*p* = 0.015). Among specific procedures, PML reconstruction, annuloplasty ring implantation, loops and cleft plicature were more commonly performed using MIS than CS, while resection was more likely to be performed in CS than in MIS patients (Table 3). The overall rate of ‘short-term’ successful valve repair was significantly higher with MIS than CS (*p* < 0.001).

**Table 3:** Procedural details

	MIS (N = 422)		CS (N = 422)		
	n/N or n	% or Mean ± SD	n/N or n	% or Mean ± SD	*p* value
*Mitral valve repair*	378/422	89.6	354/422	83.9	**0.015**
AML reconstruction	60/378	15.9	75/354	21.2	0.064
PML reconstruction	280/378	74.1	244/354	68.9	0.123
Annuloplasty ring^a^	377/378	99.7	350/354	98.9	0.203
Open (Cosgrove)	156/377	41.4	190/349	54.4	**< 0.001**
Closed (CE Physio/Physio II)	219/377	58.1	150/349	43.0	**< 0.001**
Other types	2/377	0.5	9/349	2.5	
Resection	93/378	24.6	199/354	56.2	**< 0.001**
Loops	186/378	49.2	65/354	18.4	**< 0.001**
Cleft plicature	104/378	27.5	26/354	7.3	**< 0.001**
*Rate of ‘short-term’ successful repair*^b^	363/378	96.0	269/354	76.0	**< 0.001**
*Mitral valve replacement*	57/422	13.5	152/422	36.0	**< 0.001**
Planned	44/57	77.2	68/152	44.7	** < 0.001**
MV replacement after repair failure	13/57	22.8	84/152	55.3	** < 0.001**
Biological	41/57	71.9	131/152	86.2	**0.016**
Mechanical	16/57	28.1	21/152	13.8	
*Concomitant procedures*					
Cryoablation	143/422	33.9	80/422	19.0	**< 0.001**
LAA closure	84/422	19.9	167/422	39.6	**< 0.001**
Concomitant TVR	29/422	6.9	111/421	26.4	**< 0.001**
PFO closure	40/422	9.5	25/422	5.9	0.053
ASD closure	3/422	0.7	9/422	2.1	0.081
Myxom	1/422	0.2	3/422	0.7	0.624
*Times*					
Procedure time (min)	422	192.0 (171.0–225.0)	422	185.0 (161.0–218.3)	**0.002**
CPB time (min)	422	133.0 (114.0–159.3)	422	101.0 (86.0–125.3)	**< 0.001**
X-clamp time (min)	422	80.0 (66.0–98.0)	422	71.0 (59.0–90.3)	**< 0.001**
Length of intubation (h)	422	10.0 (8.0–13.0)	422	12.0 (9.0–16.0)	**< 0.001**
Length of ICU (h)	422	26.0 (22.0–48.0)	422	24.0 (21.0–64.0)	0.421
Length of hospital stay (d)	422	10.0 (9.0–12.0)	422	11.0 (8.0–17.0)	**0.001**

*p* values that are statistically significant are highlighted in bold

*AML* anterior mitral valve leaflet, *ASD* atrial septal defect, *CPB* cardiopulmonary bypass, *ICU* intensive care unit, *LAA* left atrial appendage, *MV* mitral valve, *PFO* patent foramen ovale, *PML* posterior mitral valve leaflet, *SD* standard deviation, *TVR* tricuspid valve repair

^a^For one patient in the CS group no details for the ring were available. ^b^ Defined as MV repair without conversion to MV replacement and hospital survival

MV replacement was more common among CS patients than MIS patients. Planned (initial) valve replacement was performed in 16.1% of CS patients versus 10.4% of MIS patients (*p* = 0.015). MV replacement after a failed repair was substantially more likely to be performed via CS than MIS (*p* < 0.001). Biological valves were more common in valve replacement procedures performed via CS compared with those done using MIS (*p* < 0.001).

Among concomitant procedures, cryoablation was more common in patients treated via MIS compared with those treated with CS (*p* < 0.001), while LAA closure and tricuspid valve repair were more common in those treated via CS compared with MIS ( both *p* < 0.001).

Median procedure time was 7.0 min longer with MIS than CS (*p* = 0.002), cardiopulmonary bypass time was longer by 32.0 min (*p* < 0.001) and x-clamp time was longer by 9.0 min (*p* < 0.001). However, median (IQR) intubation time was shorter with MIS than CS (*p* < 0.001). There was no significant difference in the length of ICU stay between the groups, but the length of hospital stay was 1.0 day shorter in the MIS group than the CS group (*p* = 0.001).

Among patients who underwent MIS, conversion to sternotomy was necessary in 12 cases (2.8%).

### Procedure-related complications

Table 4 summarises procedure-related complications. The rates of atrioventricular block (grade III), atrial fibrillation (AF), pericardial tamponade, pneumonia, pleural effusion and wound infection were all significantly lower in the MIS group compared with the CS group reflected in the p-value and odds ratios (OR). The rates of pneumothorax, postoperative MV insufficiency (grade II or above) and death within 72 h did not differ between the groups. After multivariate analysis with adjustment for procedural differences between the two groups all significant differences remained except pleural effusion.

**Table 4:** Procedure-related complications

	MIS (N = 422)		CS (N = 422)		*p* value	Unadjusted OR	Adjusted OR*
	n/N	%	n/N	%			
Conversion to open sternotomy	12/422	2.8	n.a	n.a	n.a	n.a	n.a
Immediate 72 h procedural mortality	3/422	0.7	3/422	0.7	1.000	1.000 (0.201–4.983)	2.553 (0.368–17.716)
Wound infection	0	0	16/422	3.8	**< 0.001**	**n.a**	**n.a**
Pericardial tamponade	2/422	0.5	35/422	8.3	**< 0.001**	**0.053 (0.013–0.220)**	**0.049 (0.011–0.213)**
AV block grade III	11/422	2.6	48/422	11.4	**< 0.001**	**0.209 (0.107–0.408)**	**0.231 (0.111–0.482)**
Pneumonia	8/422	1.9	36/422	8.5	**< 0.001**	**0.207 (0.095–0.451)**	**0.279 (0.117–0.662)**
Pneumothorax	6/422	1.4	4/422	0.9	0.525	1.507 (0.422–5.380)	1.408 (0.322–6.159)
Pleural effusion	5/421	1.2	18/422	4.3	**0.006**	**0.270 (0.099–0.733)**	0.478 (0.156–1.468)
AF^a^	63/422	14.9	106/422	25.1	**< 0.001**	**0.523 (0.370–0.740)**	**0.589 (0.392–0.885)**

*p* values and OR that are statistically significant are highlighted in bold

^*^Adjusted for procedural differences between the two groups: MV repair, MV replacement, Cryoablation, LAA closure, concomitant TVR

^a^All patients with documented postoperative atrial fibrillation; *AF* atrial fibrillation, *AV* atrioventricular, *MVI* mitral valve insufficiency

In 12 cases (n = 2.8%) a conversion to open sternotomy became necessary during MIS. Five patients were converted because of bleeding (aortic, right ventricle, and mediastinal). Four patients had lung adhesions, one patient a funnel breast, one patient aortic valve insufficiency and one patient a mitral valve annular dissection and required sternotomy.

### Functional outcomes

Median MV gradient was significantly lower in the MIS group than the CS group immediately after surgery, and remained significantly lower over a mean of 5.3 years (MIS) and 7.7 years (CS) of follow-up (Fig. 2a). A greater proportion of patients treated via MIS had no (or trace) mitral regurgitation postoperatively compared with those treated via CS (70.6% versus 50.3%, *p* < 0.001; Fig. 2b). The difference was smaller, but still significant, after a mean of 5.3 years (MIS) and 8.0 years (CS) of follow-up (*p* = 0.047).

**Fig. 3:**
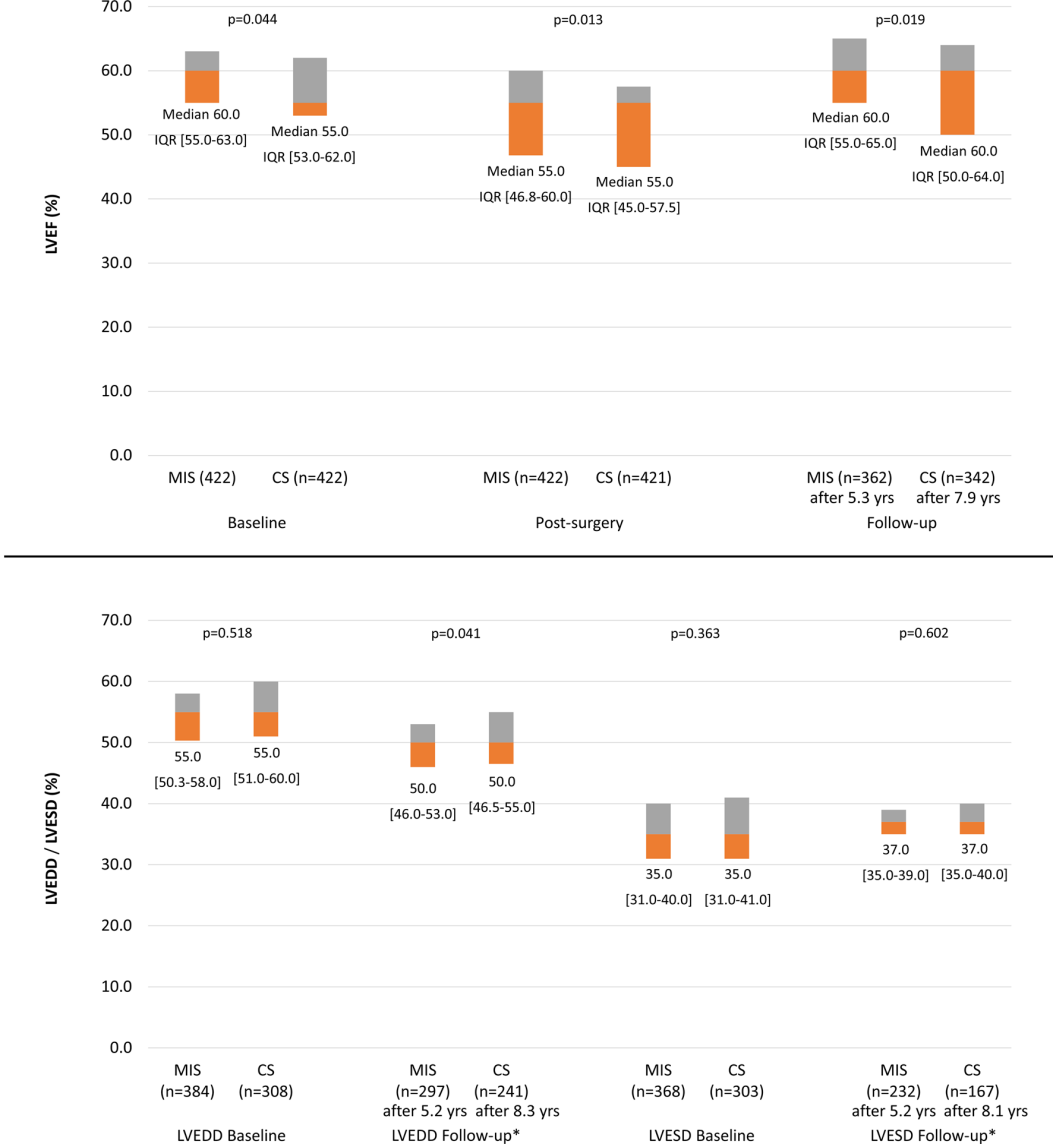
Left ventricular dimensions and function (left ventricular end diastolic diameter, LVEDD; left ventricular end systolic diameter, LVESD; left ventricular ejection fraction, LVEF). *We only used data of patients with LVEDD/ LVESD-values if they had also stated LVEDD/LVESD- baseline values to describe the course. *CS* conventional sternotomy, *IQR* interquartile range, *LVEDD* left ventricular enddiastolic diameter, *LVEF* left ventricular ejection fraction, *LVESD* left ventricular endsystolic diameter, *MIS* minimally invasive MV surgery, *MV* mitral valve

Median LVEF was lower in the CS group than in the MIS group at baseline (Fig. 3a). In the CS group, median LVEF did not change postoperatively, and had increased above baseline at long-term follow-up (after 7.9 years). In the MIS group, median LVEF decreased postoperatively, but returned to baseline after a mean of 5.3 years of follow-up. The between-group comparison favoured MIS at both timepoints (Fig. 3a). Median left ventricular end diastolic diameter (LVEDD) and left ventricular end systolic diameter (LVESD) did not differ between the groups at baseline. After 5.2 years (MIC) and 8.3 years (CS-LVEDD) and 8.1 (CS-LVESD) of follow-up, median LVEDD had decreased and median LVESD had increased in both groups; the between-group comparison of LVEDD at this timepoint favoured MIS (*p* = 0.041; Fig. 3b).

**Fig. 4:**
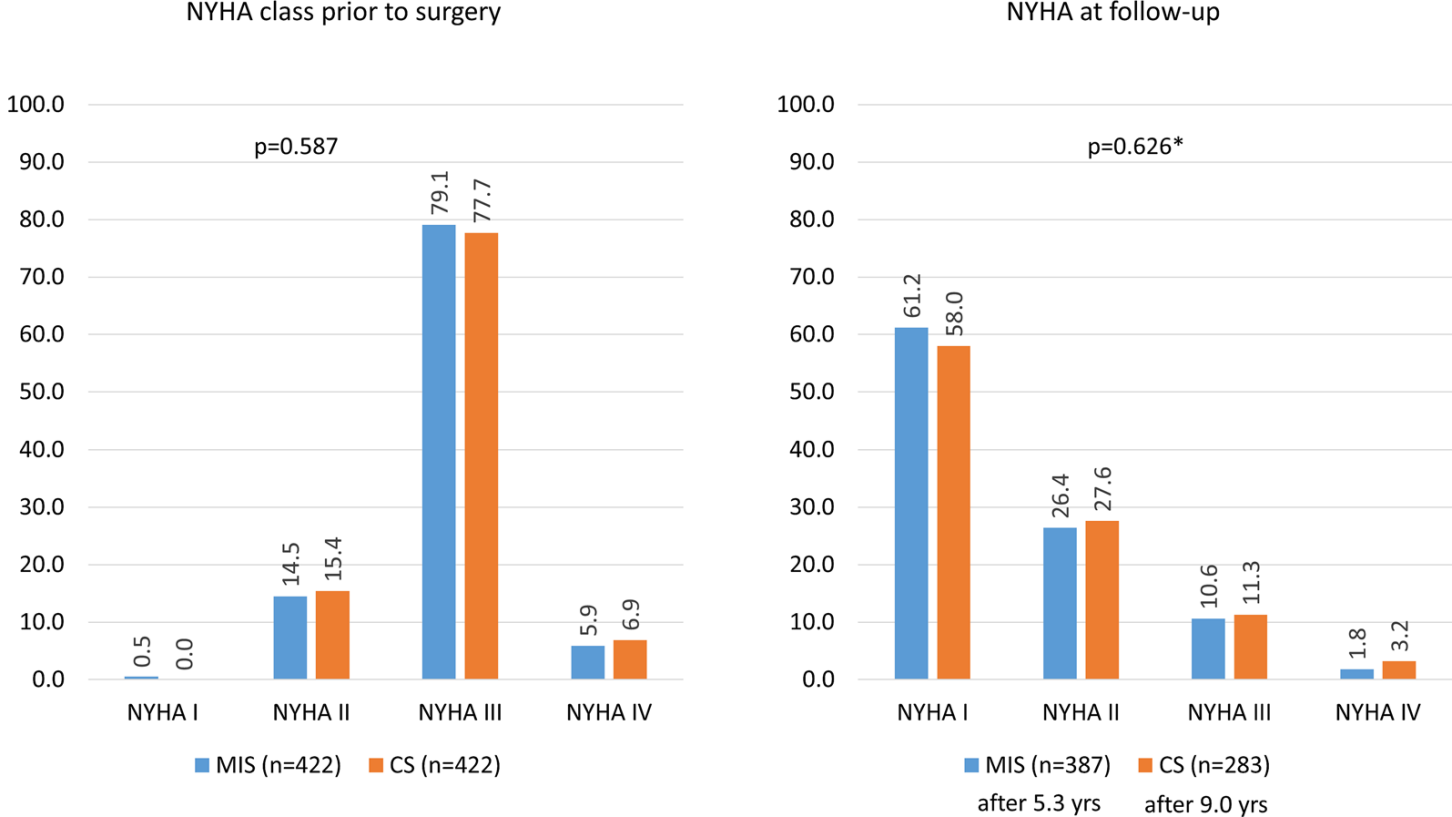
New York Heart Association (NYHA) class. *CS* conventional sternotomy, *MIS* minimally invasive MV surgery, *MV* mitral valve, *NYHA* New York Heart Association

At baseline most patients were in NYHA class III. After a mean of 5.3 years (MIC) and 9.0 years (CS) of follow-up, most patients were in NYHA class I, with no significant difference in the distribution of classes between the MIS and CS groups (Fig. 4).

These values were collected at the last follow- up visit. Due to the fact that MIS was first performed in 2009 the follow-up time for these patients is shorter than for CS patients.

### Hard outcomes

There was no difference between the groups in terms of the overall rate of death within 30 days (Table 5); however, the rate of cardiovascular death was significantly lower after MIS compared with CS (*p* = 0.015). After multivariate analysis with adjustment for main procedural differences between the two groups this difference was no longer significant. Implantation of a pacemaker was required less often after MIS than CS (*p* < 0.001), and acute renal failure was less common after MIS (*p* < 0.001). These differences between the groups remained significant in the multivariate analysis. There were no significant between-group differences with respect to rates of stroke, myocardial infarction or repeat MV surgery.

**Table 5:** 30-day outcomes

	MIC (N = 422)		CS (N = 422)		*p* value	Unadjusted OR	Adjusted OR*
	n/N	%	n/N	%			
Death	11/422	2.6	19/422	4.5	0.137	0.568 (0.267–1.208)	0.893 (0.370–2.156)
Cardiovascular death	5/422	1.2	16/422	3.8	**0.015**	**0.304 (0.110–0.838)**	0.402 (0.130–1.247)
Non-CV death	6/422	1.4	3/422	0.7	0.505	2.014 (0.500–8.108)	4.839 (0.952–24.595)
Stroke	9/422	2.1	16/422	3.8	0.155	0.553 (0.242–1.266)	0.524 (0.204–1.342)
Acute renal failure	9/422	2.1	50/422	11.9	**< 0.001**	**0.162 (0.079–0.334)**	**0.221 (0.101–0.482)**
Myocardial infarction	3/422	0.7	0	0	0.249	n.a	n.a
Pacemaker implantation	14/422	3.3	47/422	11.2	**< 0.001**	**0.273 (0.148–0.504)**	**0.307 (0.155–0.608)**
Repeat MV surgery	1/422	0.2	3/422	0.7	0.624	0.332 (0.034–3.202)	0.825 (0.059–11.464)

*p* values and OR that are statistically significant are highlighted in bold

^*^Adjusted for procedural differences between the two groups: MV repair, MV replacement, Cryoablation, LAA closure, concomitant TVR; *CV* cardiovascular, *MV* mitral valve

**Fig. 5:**
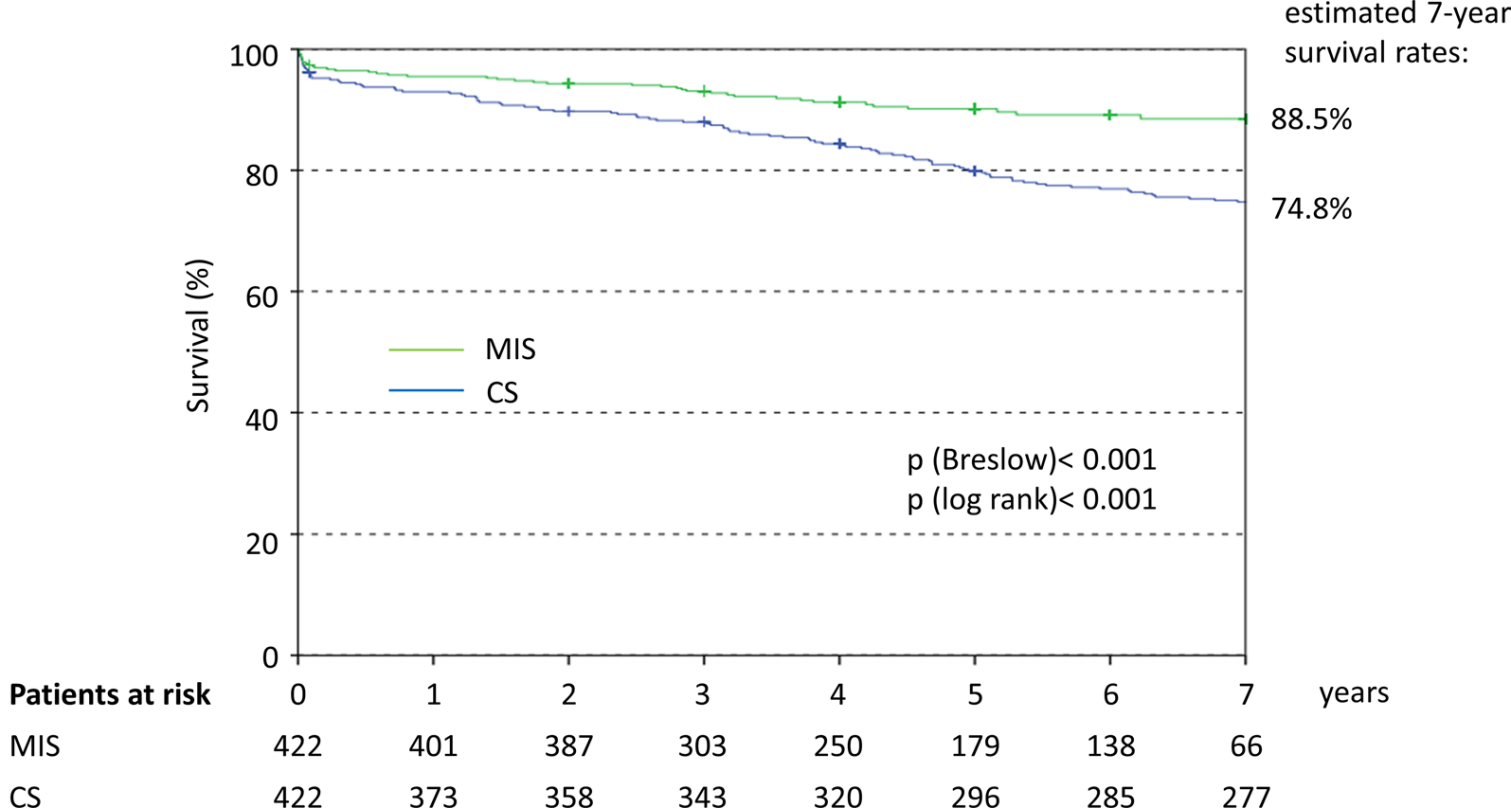
Kaplan Meier curve for long-term survival. HR calculated by Cox regression: 0.443 (95% CI 0.308–0.637) and after adjustment for procedural differences (MV repair, MV replacement, Cryoablation, LAA closure, concomitant TVR) 0.475 (95%CI 0.317–0.713) in favour of MIS. *CS* conventional sternotomy, *LAA* left atrial appendage, *MIS* minimally invasive MV surgery, *MV* mitral valve

Long-term survival is summarised in Fig. 5. The estimated 7-year survival rate was significantly better after MIS compared with CS, HR = 0.443 (95% CI 0.308–0.637) in favour of MIS and remaining after adjustment for procedural differences.

## Discussion

The results show that at our center, a higher rate of ‘short-term’ successful MV repair, a lower rate of cardiovascular death within the first 30 days, and better long-term survival, was observed with MIS compared with CS. We also observed fewer procedure-related complications, better functional outcomes and a shorter length of hospital stay, as well as lower rates of pacemaker implantation and acute renal failure within 30 days, with MIS. MIS and CS did not differ with respect to the incidence of stroke, myocardial infarction and repeat MV surgery.

MIS has been shown to be associated with low mortality and good postoperative, mid-term and long-term results [8, 9, 12, 13, 18, 20–22]. The use of MIS instead of CS for MV surgery has increased in recent years. At our center, MIS was introduced in 2009, and the majority of patients undergoing MV procedures are now treated using this approach. CS is more likely than MIS to be used in high-risk patients and to compare outcomes after MV procedures performed using these two approaches we excluded patients that were not eligible in principle for MIS and incorporated propensity-score matching to control for differences in preoperative patient and risk profiles.

MV repair was the most common procedure, performed in more than 80% of patients irrespective of the access route. The rate of ‘short-term’ successful repair, defined as MV repair without conversion to MV replacement and hospital survival, was 96.0% in the MIS group and 76% in the CS group (*p* < 0.001). This is in contrast to meta-analyses which found that repair rates were similar with MIS and CS [4, 5]. In our study, however, as there was no difference in the rate of immediate procedural death, the difference observed is because of the higher rate of conversion from repair to replacement in the MIS group. Interestingly, MV replacement was planned upfront in the CS group more often than in the MIS group and the difference (95 patients) accounts for this. It appears, therefore, as if repair was usually intended in MIS patients, but repair rates were quite similar between the two groups. The median gradient was higher in the CS at baseline compared to the MIS group and this difference largely persists after treatment, albeit at a lower level. The conversion rate to sternotomy from MIS was low (2.8%), which is consistent with previous studies (1–2.6%) [23, 24].

Operating time, cardiopulmonary bypass time and cross-clamp time were longer with MIS than with CS, as has been reported previously [6, 8, 10–12, 14, 18, 20] Despite the longer procedural time, there was no difference in the length of ICU stay, and the overall length of hospital stay was shorter in the MIS group. Previous studies have also generally reported shorter ICU or hospital length of stay after MIS compared with CS [6, 8, 10–12, 14–17, 20]. The shorter hospital stay suggests postoperative recovery was quicker in MIS patients and is also consistent with the lower incidence of procedure-related complications seen after MIS compared with CS in our study. Overall, the length of hospital stay reported in this study appears to be quite high by today’s standards, but this study captured data from 2005 until 2015, and a lower length of stay would currently be expected. In addition, some healthcare systems (e.g., German healthcare) dictate a minimum length of hospital stay post-treatment for reimbursement, while other healthcare systems discharge patients as early as it is safe to do so to reduce treatment costs.

In the current study, there was no difference between MIS and CS with respect to mortality within the first 72 h post-procedure, or the overall rate of mortality within 30 days. This is consistent with most other studies, all of which evaluated all-cause mortality [3, 4, 6–8, 12].

We also evaluated long-term mortality, and after a mean of 7 years found a better survival rate among patients treated via MIS compared with CS (88.5% versus 74.8%, *p* < 0.001). Previous studies have generally found no significant difference in mid-term (1–3 year) [11, 12, 20, 22] or long-term (4–9 years) [12, 15, 18, 20, 22] survival between patients treated via MIS or CS. However, one propensity-matched study involving patients with degenerative MV regurgitation reported better survival after MIS, with 5- and 10-year survival rates of 90% and 84% compared with 85% and 70% after CS (*p* = 0.004) [13]. Our results are consistent with these values.

We did, however, find a lower rate of pacemaker implantations and less risk of acute renal failure in the MIS group. Pacemaker implantation is found to be more common in patients receiving concomitant tricuspid valve replacement [25, 26]. In our dataset, 16.0% of the patients with concomitant tricuspid valve repair versus (TVR) 8.0% of the patients with isolated MV surgery needed implantation of a pacemaker (*p* = 0.037). However, after adjustment for TVR amongst other variables in the present analysis the OR remained significant. Another propensity-matched study has reported a lower rate of dialysis for renal failure after MIS compared with CS [13], but most studies and meta-analyses have found no difference in these two outcomes between MIS and CS [4, 6, 14–16, 18–20]. Nonetheless MIS may be associated with a need for conversion to CS and we find patients with previous right lateral thoracotomy, lung adhesions and peripheral artery disease are at increased risk. Furthermore, we observed a case where the inferior vena cava was interrupted and conversion to surgery needed [27]. Finally, we gathered experience in performing MIS in patients with prior surgery of the breast, such as mammoplasty [28].

The study has several limitations. The principal ones were the retrospective nature of the analysis, the time shift with more CS being performed in the initial years and more MIS later on, the potential for a skewed group assignment based on concomitant disease having an impact on access route selection, and the definition of ‘short-term’ successful repair on study outcome. As to the first point, we considered restricting the analysis to the time of the biggest overlap and/or the second part of the time window (2010–to 2015). This would, however, increase the potential bias arising from the experience of the surgeons with the intervention (plenty for CS, less so for MIS). In addition, this would shorten the length of the follow-up considerably. As to the second point, we excluded patients from the analysis who were not eligible for either approach in principle. Our clinical standard would exclude patients with concomitant interventions CABG and/or aortic valve intervention, intervention of the ascending aorta, severe pulmonary adhesion or severe calcification of the mitral annulus and we omitted these subjects from this analysis. Furthermore, propensity score matching helped mitigate this but may not eliminate it completely and our results should be considered with this caveat. One of the 15 key baseline variable for propensity score matching was MV pathology. While the MV pathologies between the CS and MIS groups were well-matched overall, the MIS group had more patients with annulus dilatation and PML prolapse than the CS group. In an ideal scenario, patients would have been randomized for treatment and functional and mitral stenosis patients, which comprise approximately 10% of the patient populations, would have been excluded from the analysis but doing this would have limited the statistical power of the dataset. Our study defined ‘short-term’ successful repair as valve repair (without valve replacement) and hospital survival, which may introduce a bias in favor of MIS. Patients being treated with MIS are less likely to undergo repair because it would prolong the operation time and, as a result, surgeons may be more prepared to accept a small level of valve leakage. In addition to these, the analysis was based on a single center, which may limit the generalisability of the results but is usually associated with increased internal consistency.

## Conclusion

This study demonstrates that good results for MV surgery can be obtained using an MIS approach, with a high MV repair rate, low peri-procedural morbidity and mortality, and improved long-term survival. The results support the use of a minimally invasive approach as the standard therapeutic option for MV surgery.

## Data Availability

The datasets used and/or analysed during this current study are available from the corresponding author on reasonable request.

## Abbreviations

AF: atrial fibrillation
AML: anterior mitral valve leaflet
ASD: atrial septal defect
AV: atrioventricular
CABG: coronary artery bypass grafting
CCS: Canadian Cardiac Society
CPB: cardiopulmonary bypass
CS: conventional sternotomy
CV: cardiovascular
HR: hazard ratio
ICU: intensive care unit
IQR: interquartile range
LAA: left atrial appendage
LVEDD: left ventricular end diastolic diameter
LVEF: left ventricular ejection fraction
LVESD: Left ventricular end systolic diameter
MI: myocardial infarction
MIS: minimally invasive surgery
MV: mitral valve
MVI: mitral valve insufficiency
NYHA: New York Heart Association
OR: odds ratio
PFO: patent foramen ovale
PISA: proximal isovelocity surface area
PML: posterior mitral valve leaflet
SD: standard deviation
TEE: transoesophageal echocardiography
TVR: tricuspid valve repair
